# Supplementary material for: A Mutation Network Method for Transmission Analysis of Human Influenza H3N2
Source: Viruses. 2020 Oct 3;12(10):1125. doi: 10.3390/v12101125 (PMC7601908; doi:10.3390/v12101125)
Supplement: Supplementary file 1 [file viruses-12-01125-s001.pdf]

Supplementary Materials

Table S1. Predefined inter-regional transmission intensity (TI) matrix.

To / From	A	B	C	D	E	F
A	—	0.0400	0.0060	0.0320	0.0070	0.0200
B	0.0280	—	0.0080	0.0180	0.0066	0.0080
C	0.0070	0.0070	—	0.0066	0.0180	0.0240
D	0.0200	0.0160	0.0070	—	0.0140	0.0080
E	0.0070	0.0066	0.0220	0.0180	—	0.0120
F	0.0150	0.0090	0.0260	0.0120	0.0140	—

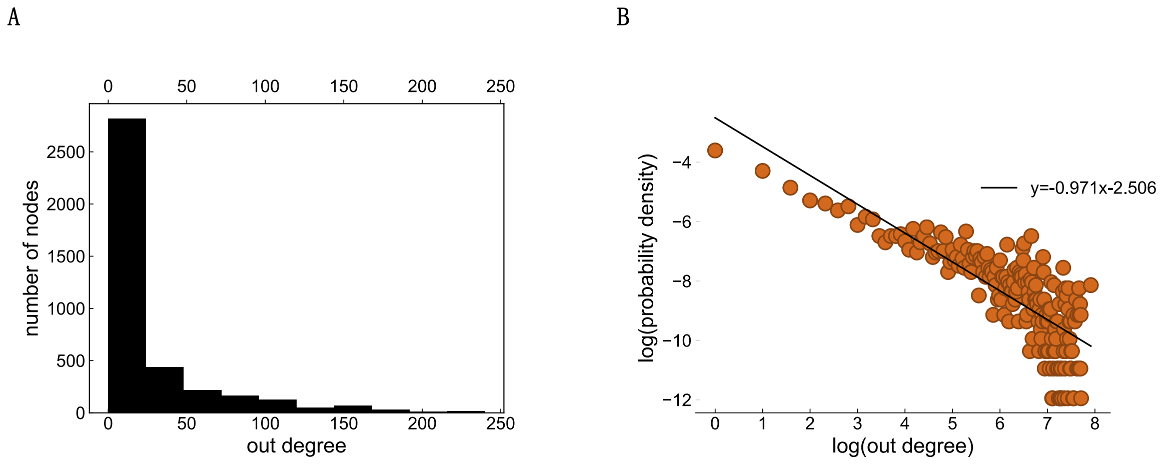

Figure S1. Power law distribution of out degree of nodes. A) Out degree distribution histogram for the mutation network constructed based on the Bedford Data. B) Scatter plot for the out degree and its probability density under the logarithmic scale. Fitted linear regression function was also given.
